# Effect of nanoporous membranes thickness in electrochemical biosensing performance: application for the detection of a wound infection biomarker

**DOI:** 10.3389/fbioe.2024.1310084

**Published:** 2024-02-23

**Authors:** C. Toyos-Rodríguez, D. Valero-Calvo, A. Iglesias-Mayor, A. de la Escosura-Muñiz

**Affiliations:** ^1^ NanoBioAnalysis Group, Department of Physical and Analytical Chemistry, University of Oviedo, Oviedo, Spain; ^2^ Biotechnology Institute of Asturias, University of Oviedo, Oviedo, Spain

**Keywords:** nanochannel, nanochannel thickness, catalase, wound infection, sensing

## Abstract

**Introduction:** Nanoporous alumina membranes present a honeycomb-like structure characterized by two main parameters involved in their performance in electrochemical immunosening: pore diameter and pore thickness. Although this first one has been deeply studied, the effect of pore thickness in electrochemical-based nanopore immunosensors has been less taken into consideration.

**Methods:** In this work, the influence of the thickness of nanoporous membranes in the steric blockage is studied for the first time, through the formation of an immunocomplex in their inner walls. Finally, the optimal nanoporous membranes were applied to the detection of catalase, an enzyme related with chronic wound infection and healing.

**Results:** Nanoporous alumina membranes with a fixed pore diameter (60 nm) and variable pore thicknesses (40, 60, 100 μm) have been constructed and evaluated as immunosensing platform for protein detection. Our results show that membranes with a thickness of 40 μm provide a higher sensitivity and lower limit-of-detection (LOD) compared to thicker membranes. This performance is even improved when compared to commercial membranes (with 20 nm pore diameter and 60 μm pore thickness), when applied for human IgG as model analyte. A label-free immunosensor using a monoclonal antibody against anti-catalase was also constructed, allowing the detection of catalase in the range of 50–500 ng/mL and with a LOD of 1.5 ng/mL. The viability of the constructed sensor in real samples was also tested by spiking artificial wound infection solutions, providing recovery values of 110% and 118%.

**Discussion:** The results obtained in this work evidence the key relevance of the nanochannel thickness in the biosensing performance. Such findings will illuminate nanoporous membrane biosensing research, considering thickness as a relevant parameter in electrochemical-based nanoporous membrane sensors.

## 1 Introduction

The raising spread of rapidly evolving illnesses (Baker et al., 2022), as well as the overcrowd of primary care centers (Sartini et al., 2022), have demonstrated the need for rapid and reliable diagnostic tools for a proper healthcare management (Bernabé-Ortiz et al., 2021). With the 50% of illnesses detected nowadays being diagnosed in purpose-built centralized laboratories (Luppa et al., 2011), the need of faster alternatives is mandatory. Traditional techniques as polymerase chain reaction (PCR) or enzyme-linked immunosorbent assay (ELISA) (Sokolenko and Imyanitov, 2018), cell culture (Opota et al., 2015), and mass spectrometry (Unlu and Abusoglu, 2022) are precise, robust and, in most cases, automated techniques, but they still lack from the decentralization and cost-effectivity demanded for a point-of-care clinical diagnosis.

Biosensing devices constitute suitable alternatives in this sense, with highly diverse materials and formulations (Campuzano and Pingarrón, 2023) that allow the detection of nucleic acids as desoxyribonucleic acid (DNA) and ribonucleic acid (RNA) or protein biomarkers. From the biosensing strategies available, nanoporous materials constitute a robust alternative, proof-of-which are commercial examples as the DNA/RNA sequencing MinION technology, based on this sensing principle (Wang et al., 2021).

Nanopore sensing relies on the monitoring of current fluctuations between two chambers filled with an electrolyte solution through a nanopore inserted inside an insulating membrane (Xue et al., 2020). The passage of an analyte through the pore reduces the current recorded in a specific signature, associated to the size, charge or sequence of the target molecule.

Nanopore sensing was initially developed making use of single protein pores (Bayley and Cremer, 2001) although the stability and size limitations of this technology, soon prompted the advancement of solid-state alternatives (Dekker, 2007) with nanopore sizes between 100 nm and 1 nm (Liu et al., 2023) and variable non-permeable materials.

All these membrane-based sensors rely on resistive pulse as sensing principle. However, the monitoring of current changes through these means has disadvantages, as the need to minimize signal-to-noise effects or the difficulty of multiplexing (Xue et al., 2020).

The combination of nanoporous membranes with alternative sensing strategies, as optical sensing (Spitzberg et al., 2019), field-effect transistors, quantum tunnelling (Ren et al., 2017) or electrochemistry (Ito and Nathani, 2022) is then a promising alternative.

In combination with electrochemistry, solid-state membranes, and more particularly nanoporous alumina membranes have been deeply used for sensing applications (de la Escosura-Muñiz and Merkoçi, 2012). This type of membranes stands out due to their ease of functionalization, large surface area, stability, and filtering properties. Nanoporous alumina membranes have a homogenous and self-ordered nanoporous structure, formed by the voltage mediated anodization of aluminium at an acidic pH, which forms a honeycomb-like structure characterized by their pore diameter and pore thickness.

Several works have previously studied the effect of this first dimension in detail (Van Den Hout et al., 2010). However, the effect of pore thickness in the analytical performance of an electrochemical-based nanopore sensor is still less known. Nanopore thickness has been previously considered in biological nanopores (Xue et al., 2020), were a reduction of the length of this dimension has been correlated with an increase in the sensitivity achieved. In solid-state membranes, thickness has been also identified as a critical factor for resistive pulse recording, as both signal (current intensity) and resolution (associated with the actual sensing region inside the nanopore) are inversely proportional to this dimension.

In this context, this work has evaluated for the first time the effect of nanoporous alumina membrane thickness in a sensing device relying in electrochemical detection. Membranes with a fixed pore diameter (60 nm) and variable pore thicknesses (40, 60, 100 μm) have been constructed through a two-step anodization process. Commercially available membranes with a diameter of 20 nm and a thickness of 60 μm have been also evaluated for comparison purposes. The analytical characteristics of these membranes have been compared developing a model immunosensor for the detection of human immunoglobulin G (HIgG) as model analyte. The principle of the developed sensor is based on the immobilization inside nanoporous alumina membranes of an antibody against HIgG. In the presence of this molecule, an immunocomplex is formed inside the channel, which hinders the diffusion of the redox indicator ferrocyanide ([Fe(CN)_6_]^4-^) through the nanochannel, changing the electrochemical signal recorded.

Optimal nanoporous membranes have been applied to the detection of a chronic wound infection biomarker as a proof-of-concept. Chronic wounds are a prevalent healthcare challenge in aging populations, affecting 1%–2% population in developed countries (Clinton and Carter, 2015). A major complication in the normal healing process of a wound is infection, that delays wound bed recovery and, if unattended, increases the risk of sepsis. A fast and accurate identification of an infection is then mandatory to stop bacteria colonization by providing a suitable antibiotic treatment. However, current identification techniques are based on first instance on visual signs of infection (Siddiqui and Bernstein, 2010) (i.e., redness, swelling, increased temperature of the wound bed, etc.) and as confirmation, gold-standard culture techniques. The long time required for these techniques to provide a result leads to the unrestrained administration of antibiotic treatments, what aggravates the appearance of multi-drug resistant microorganisms (Miethke et al., 2021; Inda-Díaz et al., 2023). The substitution of these approaches by point-of-care analytical tools, as the one proposed in this work, are desirable to tackle infection in a cost-effective, fast, and accurate manner.

Catalase, a hydrogen peroxidase enzyme that has been stablished as biomarker of several pathologies, including oxidative stress or chronic wound infection (Clemente et al., 2020), has been selected as target analyte. Catalase is produced by certain bacteria, including *Pseudomonas aeruginosa*, a prevalent microorganism present in chronic wounds, as well as Enterobacteriaceae or *Staphylococcus* among others (Chester and Moskowitz, 1987; Shin et al., 2008). But catalase can be also produced by the human body, and it has been related with the healing state of a wound (Rasik and Shukla, 2001), constituting a dual infection and healing biomarker. This relation has prompted the incorporation of catalase or catalase-like nanozymes (Wang et al., 2023; Xu et al., 2023) as components of wound dressing materials, pointing out the relevance of this biomolecule in wound management.

## 2 Experiments

### 2.1 Materials

High purity aluminum discs (Al 99.999%, Goodfellow, United Kingdom), (3-aminopropyl) triethoxysilane (APTES), catalase from human erythrocytes, monoclonal anti-catalase antibody, N-(3-Dimethylaminopropyl)-N′-ethylcarbodiimide hydrochloride (EDC), Human IgG >95% (HPLC grade), polyclonal anti-Human IgG antibody, N-Hydroxysulfosuccinimide sodium salt (sulfo-NHS), potassium ferrocyanide K_4_[Fe(CN)_6_], (2-(N-morpholino) ethanesulfonic acid) (MES) and Tris (tris(hydroxymethyl) aminomethane)-HCl (Tris-HCl) were purchased from Sigma-Aldrich (Spain). All acids required for the anodization process were purchased from VWR International Eurolabs (Spain).

Unless otherwise stated, all buffer solutions were prepared in ultrapure water (18.2 MΩ cm @ 25°C) obtained from a Millipore Direct-Q^®^ 3 UV purification system from Millipore Ibérica S.A (Spain).

Commercial Whatman^®^ AnodiscTM filter membranes (13 mm diameter, 60 μm thickness, 20 nm pore) used as control were obtained from VWR International Eurolabs (Spain). As working electrode, indium tin oxide/poly(ethylene terephthalate) (ITO/PET) sheets (with a surface resistivity of 60 Ω/sq) were obtained from Sigma-Aldrich (Spain). Silver/silver chloride from CH Instruments, Inc. (United States) and platinum wire from Alfa Aesar (United States) were used as reference and counter electrode respectively.

### 2.2 Instruments

The surface functionalization of the obtained nanoporous alumina membrane was performed on a Savannah 100 thermal atomic layer deposition (ALD) reactor (Cambridge Nanotech, Waltham, MA, United States). Characterization of the membranes was performed through scanning electron microscopy (SEM) using a MEB JOEL-6100 (Japan) operated at 20 kV. Electrochemical measurements were performed inside a customized methacrylate electrochemical cell with a PalmSens 3 potentiostat (PalmSens BV, Netherlands) controlled by a smartphone via Bluetooth.

### 2.3 Nanoporous alumina membranes preparation and functionalization

Nanoporous alumina membranes were obtained following a previously published procedure (Cuevas et al., 2023). Briefly, a highly pure aluminum disk (composed of Al 99.999% with a size of 0.5 mm in thickness and 25 in diameter) was cleaned with isopropanol and ethanol for further electropolishing with a 25% perchloric acid in ethanol solution. The aluminum was then anodized following a two-step anodization process, using a 0.3 M oxalic acid solution as electrolyte, and applying an anodization voltage of 40 V. The first anodization step was applied for 24 h at 0°C-1°C. The anodization time was modified to adjust the thickness of the resulting membranes. After that, the resulting Al template was washed to remove the aluminum oxide layer with an acidic solution of chromium trioxide (CrO_3_) and phosphoric acid (H_3_PO_4_) at 35°C for 24 h. A second anodization step was then performed also applying a 40 V potential and adjusting the time of the anodization step to obtain a nanoporous membrane thickness of ∼40, 60 or 100 µm. The membrane surface was protected by depositing a layer of poly(methyl methacrylate) (PMMA). A last cleaning step was then performed using an aqueous mixture of hydrochloric acid (HCl) and copper (II) chloride (CuCl_2_) to remove a 1 cm^2^ area of remaining Al at the bottom layer. Then, a wet chemical etching was applied using an aqueous solution of H_3_PO_4_ 5% at room temperature for 120 min to open the formed pores. Afterwards, the PMMA layer was dissolved in acetone, thus exposing the porous structure of the membrane. Finally, the pore diameter was adjusted by widening them through chemical etching in a 5% aqueous solution of H_3_PO_4_ at room temperature for 1 h.

The obtained nanoporous membranes were later functionalized using ALD by pulsing water vapor and APTES while heating the substrates at 150°C.

### 2.4 Antibody immobilization and HIgG detection using membranes with different thickness

After functionalizing the inner walls of the nanoporous alumina membranes with amine groups through ALD, 30 μL of a solution of 5 mM EDC/sulfo-NHS in MES pH 5, containing a concentration of 1,000 μg/mL (highly excess) of monoclonal anti-HIgG antibody was placed on top of the nanoporous membrane and left at room temperature for incubation for 2 h. The mechanism of antibody immobilization is schematized in Supplementary Figure S1. After this time, membranes were gently washed with Tris-HCl 10 mM pH 7.40 buffer to remove the non-immobilized antibodies. Nanopores blockage obtained after the antibody immobilization step was measured at this point for each modified membrane using a 10 mM K_4_[Fe(CN)_6_] redox indicator solution in 10 mM Tris-HCl pH 9.0 solution (see Section 2.5). After this measurement, membranes were gently washed with Tris-HCl 10 mM pH 7.40 buffer and measured again without the addition of further redox indicator, confirming that there was no remaining solution inside the membrane. Then, 30 μL of solutions containing increasing concentrations of HIgG (100, 500 and 1,000 ng/mL) were placed on the membranes and left for incubation at room temperature during 1 h, following a previously optimized protocol for this analyte (de la Escosura-Muñiz and Merkoçi, 2010). After incubation, membranes were washed again and measured using a 10 mM K_4_[Fe(CN)_6_] redox indicator solution in 10 mM Tris-HCl pH 9.0.

This protocol was followed for the self-prepared membranes with a fixed pore diameter of ∼60 nn and variable pore thicknesses (40, 60, 100 μm) and for the commercial ones with a pore diameter of 20 nm and a pore thickness of 60 μm.

### 2.5 Electrochemical measurements

ITO/PET electrodes were hydrolysed by immersion in a solution of H_2_O:NH_3_:H_2_O_2_ (17:3:1) for 20 min, followed by washing in acetone, isopropanol, and water and finally letting dry. Later, the modified electrodes were electrochemically characterized by cyclic voltammetry (CV) (scan range: −0.3 V to +0.8 V; step potential: 10 mV, scan rate: 50 mV/s) in 10 mM K_4_[Fe(CN)_6_]/0.1 M Tris-HCl at different pH values to confirm that the applied hydrophilization treatment did not affect conductivity.

For the electrochemical evaluation of the blocking/unblocking of the nanoporous membranes, APTES-modified membranes were collocated on top of the hydrophilized ITO/PET electrodes and fixed inside a methacrylate block with a hole defining an electrochemical cell of 500 μL. Membranes were maintained inside the cell during the immunocomplex formation steps (see Section 2.4.), performing washing steps with the appropriate buffer inside them. Measurements were performed using a 10 mM K_4_[Fe(CN)_6_] red-ox indicator solution in 0.1M Tris-HCl (at the appropriate pH value in each case) and a three-electrode system (silver/silver chloride reference electrode, platinum wire counter electrode and ITO/PET working electrode). Differential pulse voltammetry (DPV) was used to evaluate the oxidation of [Fe(CN)_6_]^4−^ to [Fe(CN)_6_]^3−^, applying a pre-treatment at −0.1 V for 30 s and scanning between −0.1 V and +1.1 V (step potential: 10 mV, modulation amplitude: 50 mV, and scan rate: 33.5 mV/s). Measurements were carried out in triplicate using a single nanoporous membrane and ITO/PET electrode, both discarded after each measurement.

### 2.6 Catalase detection

For catalase enzyme detection, anti-catalase antibodies at a concentration of 50 μg/mL were immobilized in the inner walls of nanoporous alumina membranes following the same procedure as described in Section 2.4. The concentration of antibody was reduced in this case to improve the cost-effectivity of the sensor. First, the optimum pH used for measurement was evaluated, using buffer solutions of Tris-HCl 0.1M pH 7.0 and Tris-HCl 0.1 M pH 8.2, in an immunosensor containing a fixed catalase concentration of 500 ng/mL (added after anti-catalase antibody immobilization, as explained in Section 2.4). Control assays were performed in Tris-HCl 0.1 M pH 7.0 without catalase being added. For the quantification of catalase, increasing catalase concentrations ranging from 50 to 500 ng/mL were added to different membranes and measured using a measurement buffer solution of Tris-HCl 0.1 M pH 7.5, as optimum pH value.

### 2.7 Spike and recovery assay in artificial wound media

To evaluate the applicability of the method in a real scenario, a spike and recovery assay was performed in artificial wound media (Galliani et al., 2020), composed of sodium chloride (124 μM), sodium bicarbonate (36.8 μM), magnesium chloride (0.831 μM), calcium chloride (2.48 μM), glucose (5 μM), lactic acid (0.010 μM) and bovine albumin (0.150 μM). The pH of the solution was adjusted to 6.7. The artificial wound media was spiked with catalase at concentrations of 100 and 500 ng/mL. After incubation, membranes were gently washed with Tris-HCl 0.1 M pH 7.5 prior to measurement in a 10 mM K_4_[Fe(CN)_6_] solution in the same buffer.

## 3 Results and discussion

### 3.1 Nanoporous membranes structural characterization and ITO/PET electrodes evaluation

Nanoporous alumina membranes with thicknesses of 40, 60 and 100 μm and a diameter size of around 60 nm were obtained following a two-step anodization process by modifying the time of the second anodization step. The rationale behind the selection of 40, 60 and 100 μm as pore thickness is based on the thickness of commercially available nanoporous alumina membranes (60 μm). This type of membranes have been extensively used for sensing purpose, with parameters such as the pore diameter been deeply optimized (de la Escosura-Muñiz and Merkoçi, 2010). However, due to the fixed thickness of these membranes, this parameter was to a lesser extent studied. A nanopore thickness equivalent to the commercially available one (60 μm) was compared in this work with a lower (40 μm) and a higher (100 μm) thickness value.

**FIGURE 1:**
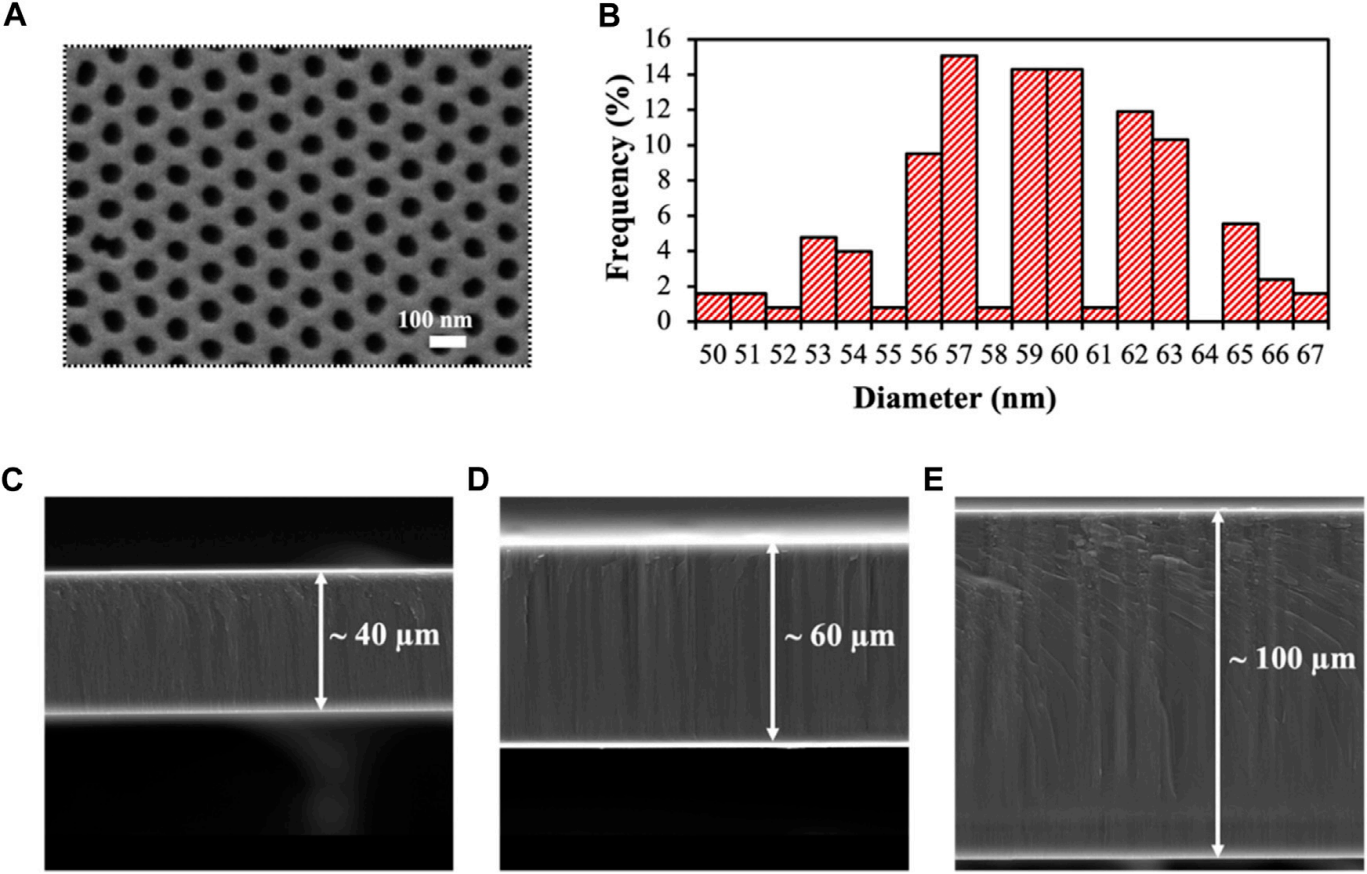
Characterization of the obtained nanoporous alumina membranes. **(A)**. SEM top-view of the self-obtained nanoporous membranes. **(B)**. Size distribution of the nanochannels diameter, showing an average size of 59 ± 4 nm. **(C, D)**. Cross-section of the self-produced nanoporous alumina membranes with thicknesses of 40 **(C)**, 60 **(D)** and 100 μm **(E)**.

The obtained nanoporous alumina membranes were characterized, both top (Figure 1A) and cross-section (Figures 1C–E) by SEM. Membranes presented a homogeneous honeycomb-like structure of an average diameter size of 59 ± 4 nm for the 40 thickness membranes (Figure 1B), maintained for all the thicknesses tested (Supplementary Figure S2). The spherical shape of the obtained nanopores contrasts with the irregularities observed in commercial filtering alumina membranes (Supplementary Figure S3) with a nanopore diameter of 20 nm, making them more suitable for their use in biosensing. Commercial membranes of 20 nm diameter sized were selected in this case as control as the diameter size is the closest available to the membranes obtained in this case, being other commercial options of diameters of 100 and 200 nm.

**FIGURE 2:**
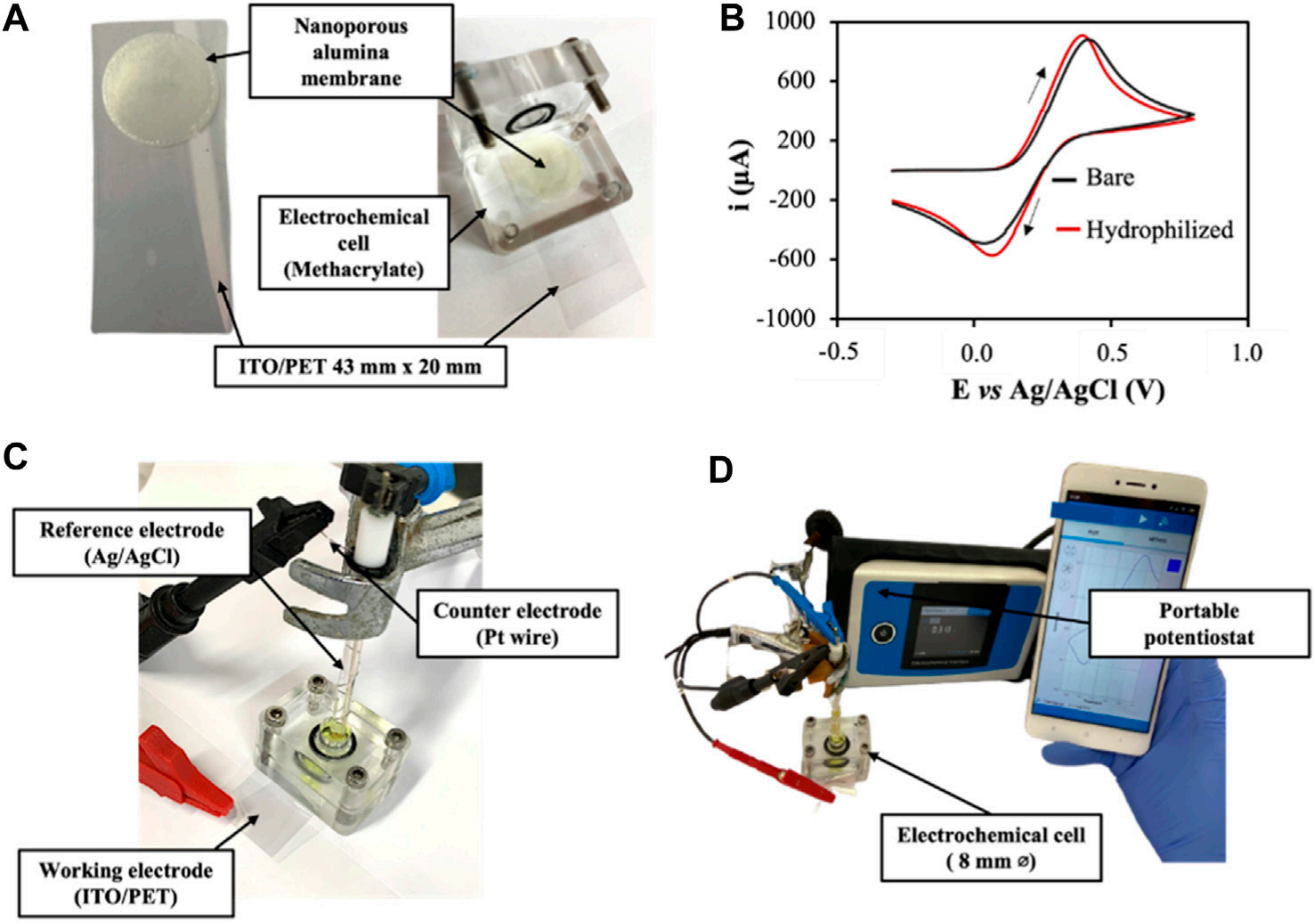
**(A)**. Picture of the nanoporous membranes integrated on top of an hydrophilized ITO/PET electrode. **(B)**. Electrochemical characterization of bare ITO/PET electrodes and hydrophilized ITO/PET electrodes. Cyclic voltammograms recorded from −0.4 to +0.8 V (vs. Ag/AgCl) in [Fe(CN)_6_]^4−^/Tris-HCl 0.1M pH 7.2. Scan rate:50 mV/s; step potential: 10 mV. **(C)**. Picture of the electrochemical cell assembling. **(D)**. Picture of the complete set-up, including the portable potentiostat controlled by a smartphone.

To increase the integrability and portability of the developed sensor, membranes were incorporated on top of a hydrophilized ITO/PET electrode (Figure 2A). The effect of the hydrophilization pretreatment over the electrochemical behavior of the ITO/PET electrode was previously evaluated by recording cyclic voltammograms in [Fe(CN)_6_]^4−^/Tris-HCl 0.1 M pH 7.2, and comparing them with the obtained for an unmodified electrode (Figure 2B). No variation was observed neither in the anodic/cathodic peak potentials nor in the current peak intensity, confirming that the hydrophilization treatment applied did not affected the electrode performance. ITO/PET electrodes were selected over alternative electrode materials, as screen-printed carbon electrodes (SPCEs) as they are flexible and biocompatible materials that also increase the stability of the measurements (by being just the working electrode covered by the membrane) (Figure 2C), increasing the reproducibility of the system. The complete set-up is shown in Figure 2D, consisting on the anchoring of the nanoporous alumina membrane and the ITO/PET electrode inside a methacrylate chamber, that clamps both pieces without generating noticeable air gaps.

### 3.2 Evaluation of membrane thickness effect in the nanochannel blockage produced by the immunocomplex

**FIGURE 3:**
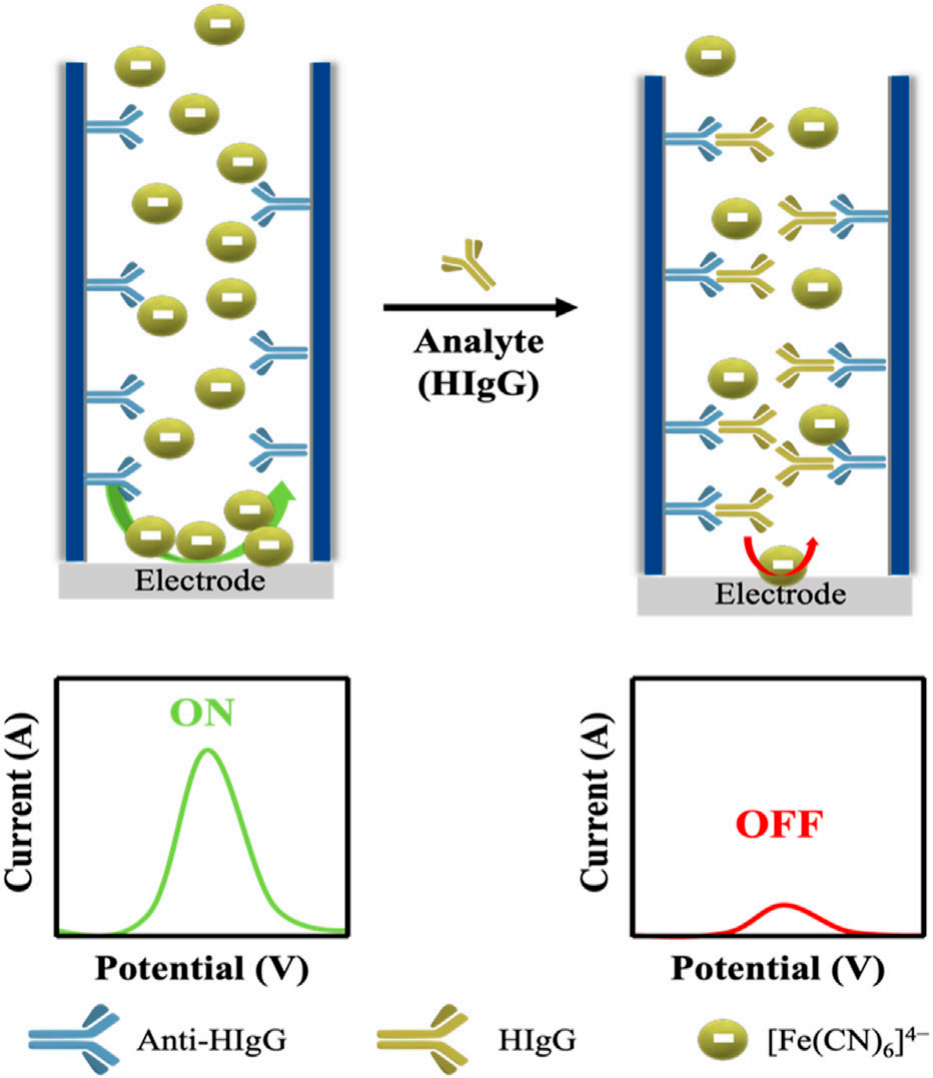
Schematic representation of the sensing principle for the immunosensor for the detection of HIgG. When anti-HIgG antibodies are immobilized inside the pore (left side), the passage of a redox indicator solution ([Fe(CN)_6_]^4-^) is slightly reduced, leaving the channel open, what leads to a high current recorded. In the presence of the target analyte, in this case HIgG, the capturing of this molecule inside the nanopore is associated to a decrease in the current recorded due to a closure of the channel.

To evaluate how the thickness of the nanoporous membranes affects the sensitivity of the (bio)analytical platform developed, an immunosensor for the detection of HIgG was constructed through the immobilization of anti-HIgG antibodies in the inner walls of the nanochannels (Figure 3). The principle of the developed platform is based on the specific capturing of HIgG inside the nanochannels, what blocks the passage of the red-ox indicator [Fe(CN)_6_]^4-^ to the electrode, reducing the voltammetric signal associated to the oxidation of this molecule.

HIgG has already been detected using commercially available nanoporous alumina membranes (de la Escosura-Muñiz and Merkoçi, 2010). In such work, the authors observed that the diameter of nanoporous membranes affected the blockage, being able to detect lower concentrations of HIgG with membranes of 20 nm of pore diameter than with those of 200 nm. However, the effect of membranes thickness has been hardly studied in these solid-state membranes.

Membranes with three different nanochannel thicknesses (40, 60 and 100 μm) and an average pore diameter of 60 nm have been fabricated and evaluated. The performance of these membranes has been compared to those of commercial membranes with 60 μm thickness and 20 nm diameter size. Since commercial diameters of 60 nm, highly desirable for an accurate comparison are not available, we selected those of 20 nm since this small size is what offers a better sensitivity (de la Escosura-Muñiz and Merkoçi, 2010). Membranes were tested for HIgG at concentrations 100, 500 and 1,000 μg/mL.

To simplify and standardize the results, the degree of blockage in the investigations reported in the following sections has been defined as the index of HIgG current blockage in percentage (Eq. (1)):

$$\text{Index of HIgG Current Blockage }\left({∆I}_{\text{HIgG}}\right)\;\left(\%\right)=\left(\frac{I_{0}\;\text{antibody}\;\text{modified}\;\text{membranes}‐\;I_{HIgG}\;\text{immunocomplex}}{I_{0}\;a\text{ntibody}\;\text{modified}\;\text{membranes}}\right)\;x\;100$$ (1)

where I correspond to the peak current value of the voltammetric oxidation of the redox indicator used.

The mentioned index was calculated for each membrane by measuring the current just after the antibody immobilization step (corresponding to I_0_ antibody-modified membranes) and after the formation of the immunocomplex (I_HIgG_ immunocomplex). This strategy, compared to the use of individual membranes for each step (Toyos-Rodríguez et al., 2023), increases the reproducibility of the method while facilitates manipulation, what increases the potential of the sensor for point-of-care applications.

Measurements were performed at a pH of 9.0, at which both anti-HIgG antibody and HIgG were expected to be negatively charged (Tang et al., 2021). The equal charge of the redox indicator [Fe(CN)_6_]^4-^ and the biomolecules inside the channel leads to the appearance of a repulsion effect that hinders the passage of the redox indicator, increasing the electrostatic blockage of the electrochemical signal obtained. This variable has been previously reported as a relevant factor in electrochemical-based nanopore sensors (Wang et al., 2010).

Apart from the electrostatic blockage owing to the charges inside the nanopore, steric effects, associated with the dimensions of the nanopore, contribute to the global blockage obtained. In particular, the influence of the membranes thicknesses in the steric blockage applied for the detection of HIgG is studied in this work for the first time.

**FIGURE 4:**
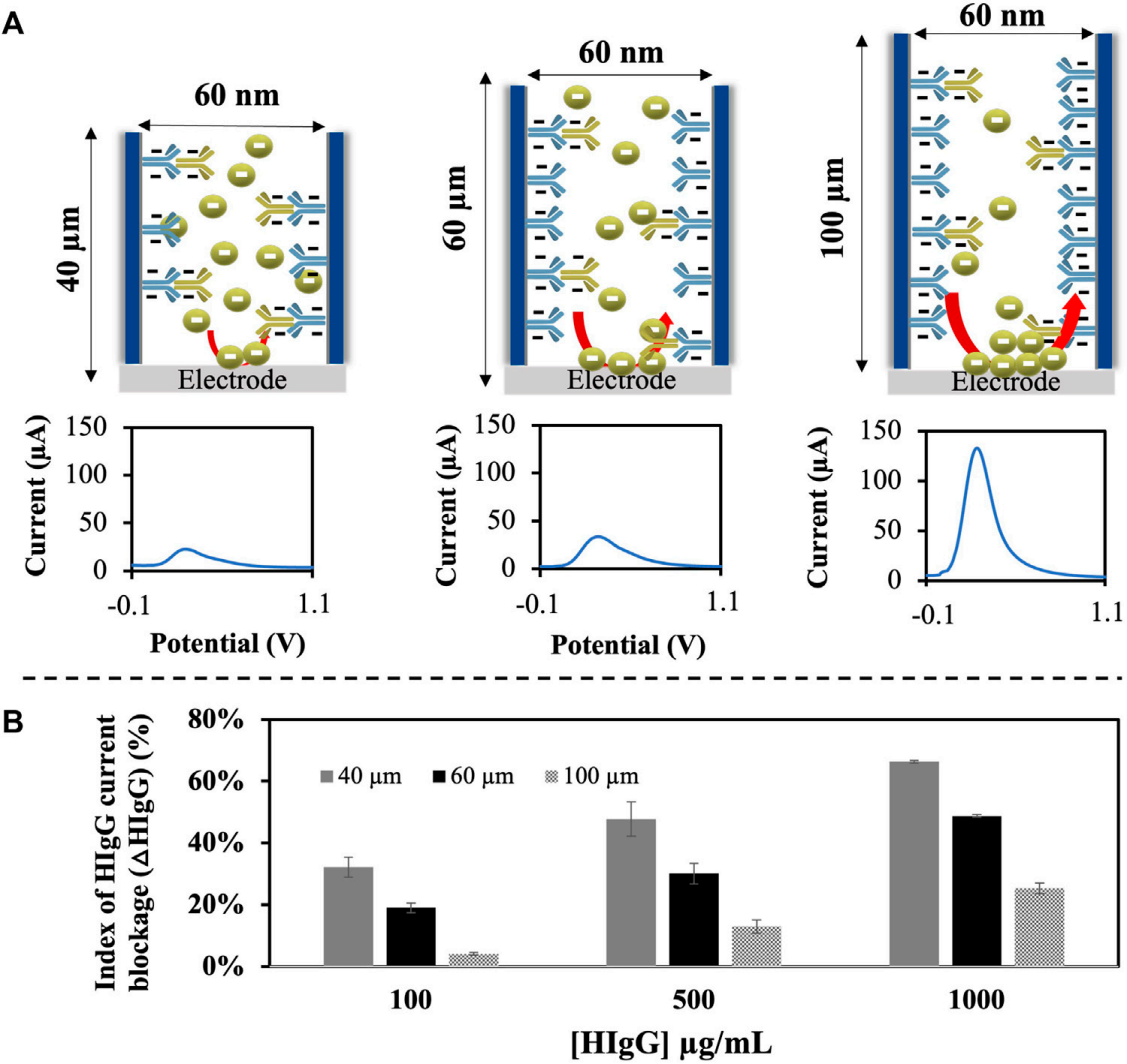
Study of the effect of membrane thickness in the sensitivity of an HIgG immunosensor. **(A)**. Schematic representation of the different membranes used with increasing thickness and different diameters, together with representative voltammograms obtained with differential pulse voltammetry (DPV) for each case at a HIgG concentration of 100 μg/mL. **(B)**. Results obtained represented as the mean and SD of the index of HIgG current blockage (ΔHIgG) (%) per each concentration studied. ΔHIgG was calculated considering the voltammetric signal, associated to the oxidation of [Fe(CN)_6_]^4-^ to [Fe(CN)_6_]^3-^ recorded through DPV with a pre-treatment at −0.1 V for 30 s and, a scan range of −0.1 V to +1.1 V (step potential: 10 mV, modulation amplitude: 50 mV, and scan rate: 33.5 mV/s).

As shown in Figure 4A, the analytical signal, corresponding to the oxidation of [Fe(CN)_6_]^4-^ to [Fe(CN)_6_]^3-^, for a fixed amount of HIgG (100 μg/mL) decreases with the membrane thickness. This behavior suggests that for the thinner membranes all the antibodies are bound to antigens, leaving a narrow path for the redox indicator ions diffusion. However, as the thickness increases, more antibodies are free leading to a wider space for the ions passage.

This behavior was also evaluated for different concentrations of HIgG, giving the results in terms of index of current blockage (Figure 4B). As can be observed, values of 32% are reached for membranes with a thickness of 40 μm for the lowest HIgG concentration tested (100 μg/mL) compared to a 4% in membranes with a 100 μm thickness. These results point out that the reduction of the nanoporous membrane thickness is directly related with the blockage obtained and hence the performance of sensors developed using this material as platform. In other words, for the thinner membranes lower amounts of antigen are enough to block the nanochannel, leading to a better sensitivity. Moreover, in all cases a linear relationship between the index of current blockage and the concentration of HIgG is obtained. The analytical characteristics obtained with the self-prepared nanoporous alumina membranes with variable thicknesses are shown at Table 1. Commercial membranes with a pore diameter of 20 nm and a thickness of 60 μm were also evaluated for comparison purposes.

**TABLE 1:** Analytical characteristics obtained or the detection of human IgG (HIgG) using nanoporous alumina membranes with different thicknesses.

Preparation	Thickness (μm)	Diameter size (nm)	Slope (ΔHIgG (%))	LOD (μg/mL)	r
Self-prepared	40	∼60	0.0379	24.2	0.999
	60	∼60	0.0332	158.8	0.997
	100	∼60	0.0236	62.5	0.999
Commercial	60	∼20	0.0373	152.8	0.997

As summarized in Table 1, when the sensitivity and limit-of-detection (LOD) obtained with each membrane were evaluated, results showed that membranes with a thickness of 40 μm provided a higher sensitivity (slope of 0.0379 ΔHIgG (%)) and lower LOD (calculated as three times the standard deviation of the intercepted divided by the slope) (24.2 μg/mL HIgG) compared to the analytical results provided with thicker membranes. For membranes with a thickness of 100 μm, the LOD obtained was lower than for those of 60 μm, even though they presented the lowest sensitivity.

For the commercial membranes used as a control with a diameter of 20 nm, a LOD more than 6 times higher than for self-prepared 40 μm membranes (152.8 μg/mL) was obtained, evidencing the better performance of our membranes and the key relevance of the nanochannel thickness.

Although the number of concentrations tested is reduced and a more representative HIgG calibration curve would be required to extract more accurate conclusions, results point out that controlling the thickness is also of paramount importance to improve the sensitivity of immunosensors and reduce de minimum quantity that can be differentiated through electrochemical recording.

Although the lowest LOD was achieved with the lower thickness 40 μm membranes, the obtained for 100 μm probed to be better than the one obtained for 60 μm. However, the sensitivity obtained in this second case is much lower, what also correlates with the index of current blockage achieved with these membranes (Figure 4). We believed that thickness of the membranes affects two aspects of the sensor that are directly related with the LOD achieved: 1) the physical space for antibody immobilization and 2) the distance between the upper side of the nanochannel and the electrode.

For the first one, the increase in the thickness also increases the number of antibodies that can be immobilized on the inner side of the nanochannel for a fixed excess concentration of antibody. In this sense, for nanoporous membranes with 100 μm thickness, a higher proportion of antibodies might be immobilized inside the nanochannel, facilitating the captured of the analyte of interest avoiding any steric impedance. However, the reduced thickness of 40 and 60 μm membranes also decreases the antibody immobilization rate that can be achieved. But although this aspect can increase steric hindrance, reducing antibody-antigen interaction, it also means that a lower protein concentration can completely block the nanochannel. This is probably a reason why the LOD is lower for 40 μm membranes, where a compromised situation between minimum protein concentration required for blocking and steric impedance is reached.

Additionally, a second factor, the distance between the upper side of a nanochannel and the electrode, also plays a role in the relation between thickness and LOD. Regarding this one, the larger the distance, the longer it takes to reach the electrode, introducing diffusion effects that can also alter the sensitivity and hence the LOD achieved.

### 3.3 Catalase wound infection biomarker detection

The optimal 40 μm-thickness nanoporous membranes were applied for the development of an immunosensor for the detection of catalase. Catalase is an important heme-containing enzyme that catalyzes the dismutation of hydrogen peroxide (H_2_O_2_) into O_2_ and H_2_O (Kurahashi and Fujii, 2015). Together with other enzymes as superoxide dismutase or peroxidase, catalase is implicated in wound healing, being downregulated in this process.

Catalase is also produced by most aerobic bacteria to neutralize the bactericidal effect of H_2_O_2_ (Dunnill et al., 2017). The identification of this enzyme is an easy way to determine the presence of infection inside a wound.

Considering the sizes of both catalase (∼240 kDa) (Pakhomova et al., 2009) and of the anti-catalase antibody (∼150 kDa) (Chiu et al., 2019), the detection of such enzyme is feasible using membranes containing nanopores of 60 nm.

Monoclonal anti-catalase antibodies were immobilized in the inner walls of the nanoporous membranes through carbodiimide chemistry. Although the exact isoelectric point (pI) of this antibody was not determined, mouse IgG present a pI in the range between 6.4 and 8.0 (Danielsson et al., 1988). Regarding human catalase from erythrocytes (UniProt Code P04040), the analyte of interest, it presents a theoretical pI of 6.9, thus a pH higher than this value would be desirable to maximize the blockage obtained. Moreover, catalase, as every enzyme, has an optimum pH range in between it maintains native conformation of the active site. For catalase, it is hypothesized that this value ranges between pH 6 and 8.

Considering this information, the pH of the measurement solution used for catalase detection was first optimized to maximize the electrostatic blockage obtained. Redox indicator solutions with pH values of 6.9 and 7.5 were tested for a catalase concentration of 500 ng/mL. At this pH range, the anti-catalase antibodies are positively charged (pH below their pI), what should favor the passage of the negatively charged red-ox indicator ions to the electrode. However, the introduction of negative charges inside the nanochannel should have an electrostatic repulsion effect over the [Fe(CN)_6_]^4−^ redox indicator, what would lead in a decrease in the voltammetric signal recorded.

Data has been normalized in terms of the index catalase current blockage as stated in Eq. (2):

Index of Catalase Current Blockage

$$\left({∆I}_{\text{Cat}}\right)\;\left(\%\right)=\left(\frac{I_{0}\;\text{anti}‐\text{Cat}\;\text{antibody}\;\text{modified}\;\text{membrane}\;‐\;I\;\text{Cat}\;\text{immunoassay}}{I_{0}\;\text{anti}‐\text{Cat}\;\text{antibody}\;\text{modified}\;\text{membrane}}\right)\;x\;100.$$ (2)

**FIGURE 5:**
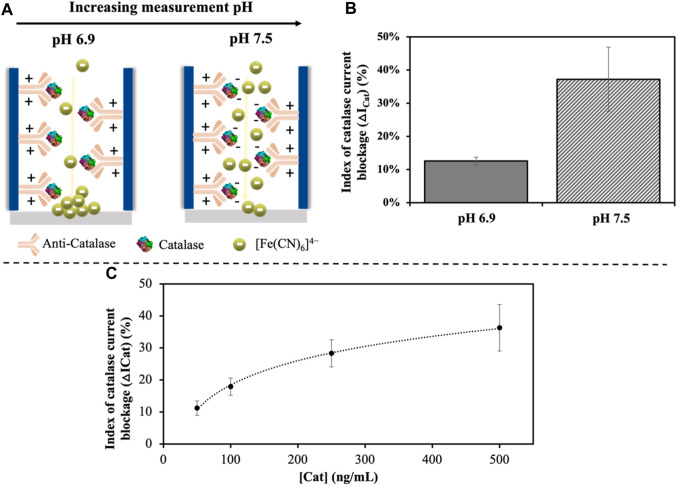
Effect of the pH used for measurement **(A)**. Representation of the electrostatic profile of the catalase immunocomplex at varying pH and how it affects the diffusion of the [Fe(CN)_6_]^4−^ red-ox indicator ions through the channel. **(B)**. Effect of the pH on the index of catalase current blockage (as defined in Eq. 2) represented by the average ±SD (n = 3). **(C)**. Effect of the catalase concentration on the index of catalase current blockage in the range of 50–500 ng/mL, showing a logarithmic relation.

According to the scheme depicted in Figure 5A, at a pH of 6.9, catalase enzyme is not charged, as this pH value almost corresponds with the pI of this molecule. As shown in Figure 5B, under these conditions, a current blockage of only 13% is obtained. However, at a pH 7.5, a high increase of ∼3 times in the index blockage (35%) is recorded, what agrees with the fact that at this pH value, catalase enzyme is negatively charged, exerting a high electrostatic hindrance inside the nanochannel. This difference observed points out the utility of this methodology not just for biomolecules detection/quantification but also for the estimation of their pI.

After the optimization of the measurement pH, catalase was determined using a [Fe(CN)_6_]^4-^ solution in Tris-HCl at a pH 7.5 as buffer solution. A logarithmic relationship between the index of catalase current blockage and the concentration of this biomolecule was observed (Figure 5C). The LOD (calculated as three times the standard deviation of the intercepted divided by the slope) obtained was of 1.5 ng/mL for a linear range of 50–500 ng/mL with a correlation coefficient (r) of 0.998, adjusted to Eq. (3):

$$\text{Peak current }\left(\mu A\right)=25.241\log\left[\text{Catalase}\right]\left(\text{ng}/\text{mL}\right)‐32.081$$ (3)

The sensor developed showed a relevant performance, with a good reproducibility (relative standard deviation (RSD) of 12% (n = 3)), and a limit of quantification (LOQ) (calculated as ten-time the standard deviation of the intercept by the slope) of 3.8 ng/mL, which is equivalent to 0.19 U/mL (∼50.000 U/mg protein). This value is in accordance with the provided by alternative techniques as the stablished spectrophotometric method of Aebi (Aebi, 1984). However, this methodology presents major drawbacks as the high concentration of H_2_O_2_ that needs to be added for the catalytic reaction to be recorded (30 mM), which can alter the activity of catalase, and the low specificity of the optical detection (at 240 nm) in biological samples (Farman and Hadwan, 2021).

The versatility of this methodology allows the detection of catalase not just as a wound management biomarker, but also as antioxidant in relation to other pathologies.

### 3.4 Spike and recovery in artificial wound fluid

The performance of the developed sensor was evaluated in an artificial wound fluid solution that mimics the composition of wound fluids in which catalase can be present (Galliani et al., 2020). By spiking an artificial wound fluid with two catalase concentrations, 100 and 500 ng/mL, the selectivity of the methodology was evaluated. The analytical signals obtained were in accordance with the ones provided in buffer solution, obtaining quantitative recoveries of 118% and 110%, respectively (Table 2). These confirms the suitability of the developed method for the detection of catalase in wound fluids, which is expected due to the low filtering properties that nanoporous alumina membranes have.

**TABLE 2:** Spike and recovery assay data in artificial wound media for catalase concentrations of 100 and 500 ng/mL.

Sample	Spiked catalase concentration (ng/mL)	Index of catalase current blockage in buffer (%)	Index of catalase current blockage in artificial wound fluid (%)	Recovery (%)
Artificial wound fluid	100	17.9	21.2	118
	500	36.3	39.9	110

## 4 Conclusion

The effect of the thickness of nanoporous alumina membranes in the sensitivity of a sensor developed using this material as sensing platform has been stated in this work. To probe this, an immunosensor based on the determination of the blockage that HIgG produces to the diffusion of the red-ox indicator [Fe(CN)_6_]^4−^ was developed. In previous works, the diameter of the nanochannels used was optimized and studied, observing that the reduction of this parameter not always favours the performance of the sensor. In this occasion, the contribution of the thickness of the nanochannel to the steric blockage was evaluated for an electrochemical-based nanoporous sensor. This work confirms that reducing this parameter increases the sensitivity while improves the LOD achieved. Our hypothesis points out that for the thinner membranes all the antibodies are bound to antigens, leaving a narrow path for the redox indicator ions diffusion. This means that lower amounts of antigen are enough to block the nanochannel, leading to a better sensitivity. However, as the thickness increases, more antibodies are free leading to a wider space for the ions passage, what is traduced in a worst sensitivity.

The reduction of the thickness to a value of 40 μm provides 6 times lower LOD than thicker commercially available nanoporous membranes (60 μm) with smaller diameter (20 nm instead of 60 nm). Overall, our work confirms that nanoporous alumina membrane thickness is as important as pore diameter and should be also considered in the use of these membranes as sensing platforms.

The optimized nanoporous membranes were applied for the detection of catalase, being able to detect this enzyme in artificial wound fluids, without sample pre-treatment, showing a low LOD. The feasibility, low cost and integrability of our system make it an ideal strategy for the rapid monitoring of not just wound infection, but also wound healing in just a few hours.

## Data Availability

The raw data supporting the conclusion of this article will be made available by the authors, without undue reservation.
