# Supplementary material for: Effect of nanoporous membranes thickness in electrochemical biosensing performance: application for the detection of a wound infection biomarker
Source: Front Bioeng Biotechnol. 2024 Feb 23;12:1310084. doi: 10.3389/fbioe.2024.1310084 (PMC10921427; doi:10.3389/fbioe.2024.1310084)
Supplement: Supplementary file 1 [file DataSheet1.docx]

**Supporting Information**

Effect of nanoporous membranes thickness in electrochemical biosensing performance: application for the detection of a wound infection biomarker

C. Toyos-Rodríguez^1,2^, D. Valero-Calvo^1,2^, A. Iglesias-Mayor^1,2^, A. de la Escosura-Muñiz^1,2*^

^1^ NanoBioAnalysis Group - Department of Physical and Analytical Chemistry, University of Oviedo, Julián Clavería 8, 33006, Oviedo, Spain

^2^ Biotechnology Institute of Asturias, University of Oviedo, Santiago Gascon Building, 33006, Oviedo, Spain

*** Correspondence:**Corresponding Author
**alfredo.escosura@uniovi.es**


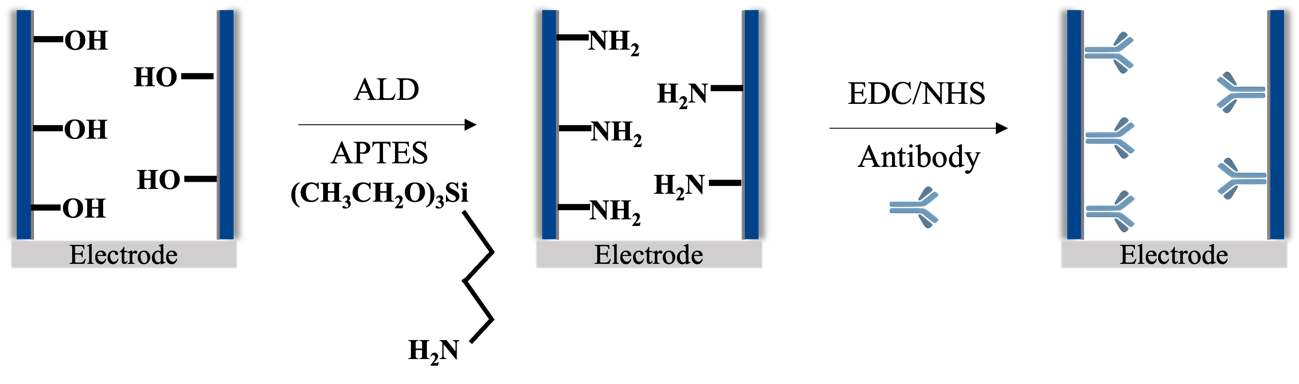


***Figure S.1****. Schematic mechanism of the immobilization of antibodies in the inner walls of nanoporous alumina membranes.*


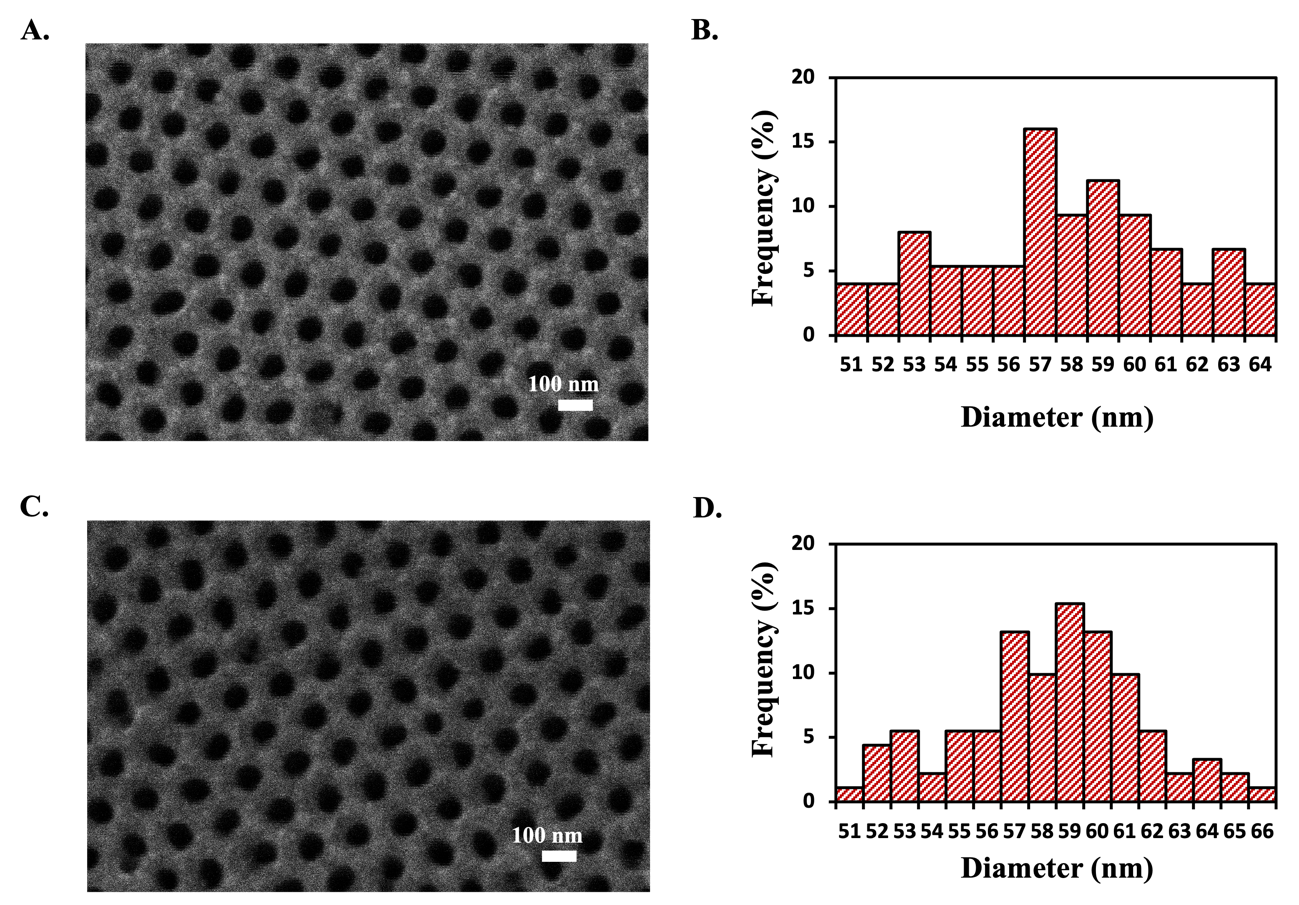


***Figure S.2.*** *Characterization of nanoporous alumina membranes of 60 μm thickness (A, B) and 90 μm thickness (C, D).*


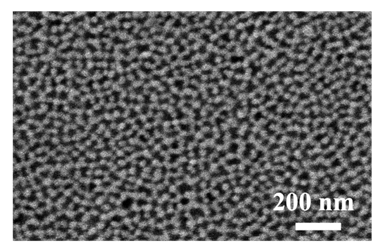


***Figure S.3****. SEM characterization of commercial nanoporous alumina membranes (top-view) with a nanopore diameter of 20 nm.*
